# G-Quadruplex-Based Fluorescent Turn-On Ligands and Aptamers: From Development to Applications

**DOI:** 10.3390/molecules24132416

**Published:** 2019-06-30

**Authors:** Mubarak I. Umar, Danyang Ji, Chun-Yin Chan, Chun Kit Kwok

**Affiliations:** Department of Chemistry, City University of Hong Kong, Kowloon Tong, Hong Kong SAR, China

**Keywords:** nucleic acids, G-quadruplex, aptamers, turn-on ligands, fluorescence, microbes

## Abstract

Guanine (G)-quadruplexes (G4s) are unique nucleic acid structures that are formed by stacked G-tetrads in G-rich DNA or RNA sequences. G4s have been reported to play significant roles in various cellular events in both macro- and micro-organisms. The identification and characterization of G4s can help to understand their different biological roles and potential applications in diagnosis and therapy. In addition to biophysical and biochemical methods to interrogate G4 formation, G4 fluorescent turn-on ligands can be used to target and visualize G4 formation both in vitro and in cells. Here, we review several representative classes of G4 fluorescent turn-on ligands in terms of their interaction mechanism and application perspectives. Interestingly, G4 structures are commonly identified in DNA and RNA aptamers against targets that include proteins and small molecules, which can be utilized as G4 tools for diverse applications. We therefore also summarize the recent development of G4-containing aptamers and highlight their applications in biosensing, bioimaging, and therapy. Moreover, we discuss the current challenges and future perspectives of G4 fluorescent turn-on ligands and G4-containing aptamers.

## 1. Introduction

**Figure 1:**
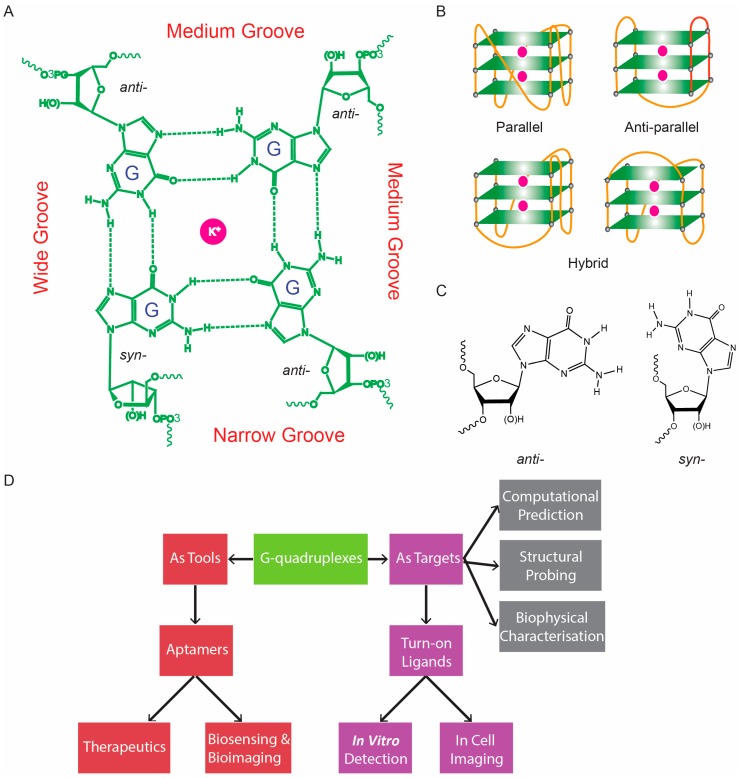
Overview of G4 structure, detection, and application. (**A**) Chemical structure of a G-quartet, showing the interactions between H-bond donors and acceptors at the Watson–Crick and Hoogsteen edges. K^+^ is located at the core of G-quartet, which can provide further stabilization. (**B**) Parallel, anti-parallel and hybrid topologies of G4, demonstrating its polymorphism. (**C**) Anti- and syn- conformations of guanosine in a G-quartet that leads to wide, narrow, and medium grooves in (A). (**D**) Review overview. Red or purple boxes are topics that will be covered, while topics in the grey boxes were reviewed elsewhere. (see references for computational prediction [46,47], structural probing [48,49], and biophysical characterization [46,50]).

Guanine (G)-rich sequences in nucleic acids have the potential to fold into structural motifs referred to as G-quadruplexes (G4s). G4s can be intra- or inter-molecularly folded, and they are formed by the stacking of G-quartets to form planar 2D structures between four guanosines by hydrogen bond interactions at their Watson–Crick and Hoogsteen edges (Figure 1A). Furthermore, a monovalent cation occupies the central cavity of the G-quartet to stabilize the structure, with a strength of stabilization in the order of K^+^ > Na^+^ > NH_4_^+^ > Li^+^ [1,2]. The formation of intra-molecular canonical G4s requires at least four regions of three consecutive Gs in a single strand, which are separated by 1–7 linking nucleotides known as loops [3]. Inter-molecular G4s are formed by G-interactions among multiple strands, from bimolecular (two strands) to tetramolecular (four strands). Generally, the stability of the G4 structure decreases as loop length increases [4,5]; therefore, it was generally thought that sequences that obey the consensus of (G_3+_N_1-7_G_3+_N_1-7_G_3+_N_1-7_G_3+_) can form G4s in the genome and transcriptome [3]. More recently, non-canonical G4s were discovered [6], such as G4s with long loops [5], bulges [7,8], 2-quartets [9], G-vacancies [10,11], duplexes [12], and triplexes [13], which broaden the sequence definition and the structural diversity of G4s. G4s are polymorphic, meaning that the G-tracts can be arranged into parallel, anti-parallel, or hybrid topologies [14,15] (Figure 1B). Guanosines can also be oriented as anti- or syn- conformations based on whether the purine rings are flipped outward or inwards with respect to the pentose sugar [14,15] (Figure 1C). Such conformational variety leads to wide, medium, and narrow grooves that describe the spatial availability of the corresponding edge of the G-quartet [14,15] (Figure 1A).

G4s play significant roles in almost every cellular event, including but not limited to DNA replication, transcription, translation, RNA metabolism, and epigenetic remodeling [16,17]. Recent studies have also suggested that G4 structures can serve as promising cancer and anti-microbe targets [18,19]. To identify such potential G4 targets, various computational methods such as QGRS Mapper [20], quadparser [3], G4 Hunter [21], G4NN [22], and Quadron [23] have been developed to predict G4 formations. Spectroscopic techniques such as circular dichroism (CD) [24], UV melting [25], mass spectrometry [26], nuclear magnetic resonance (NMR) [27], and intrinsic fluorescence [28] can also detect G4 formation based on its physical properties in a label-free manner. In addition to these biophysical methods, biochemical methods such as polymerase stop assay [29] and dimethyl sulfate (DMS) footprinting [30] can interrogate DNA G4 formation by template extension stalling and measuring the guanine nucleotide’s resistance to the attachment of a chemical probe, respectively. More recently, in vitro methods that utilize rG4-mediated reverse transcriptase stalling have been developed to interrogate rG4 in low-abundance transcripts [31], and selective 2′-hydroxyl acylation analyzed by a lithium ion-mediated primer extension (SHALiPE), and DMSLiPE [32] have been developed to map distinctive structural patterns of rG4. Several next-generation sequencing-based approaches such as G4-seq [33], G4-Chip [34], rG4-seq [35], DMS-seq [36], and G4RP [37] enable the genome-wide and transcriptome-wide profiling of G4s. Another key category for G4 detection is to use fluorogenic G4 ligands whose fluorescence is selectively enhanced when interacting with G4s. These fluorescent turn-on ligands can be used to track G4 formation both in vitro and in cells, and they are discussed in detail in this review.

Besides acting as potential targets, G4s can be used as molecular tools for diverse applications. It is worth noting that the structure of G4s has been identified in studies using combinatorial methods and the systematic evolution of ligands by exponential enrichment (SELEX) technique with the aim of developing aptamers for therapeutic and diagnostic purposes [38,39,40]. G4s provide extra chemical and thermal stability for aptamer-based therapeutics, and such aptamers have been successfully designed to target a number of HIV proteins [41,42], prion proteins [43], and anti-cancer targets [44,45]. In diagnostics, G4-containing aptamers have been widely applied to target a wide range of pathogenic proteins and small molecules to emit a fluorescence-like signal [40]. In this review, we summarize the recent development of fluorogenic G4 ligands and G4-containing aptamers, and highlight their latest applications in vitro and in cells (Figure 1D). We will also discuss current challenges and future perspectives for better detection and targeting of G4s in diverse organisms, as well as for designing and developing G4-related tools for various biological applications. 

## 2. Fluorescent Turn-on G-Quadruplex Ligand

**Figure 2:**
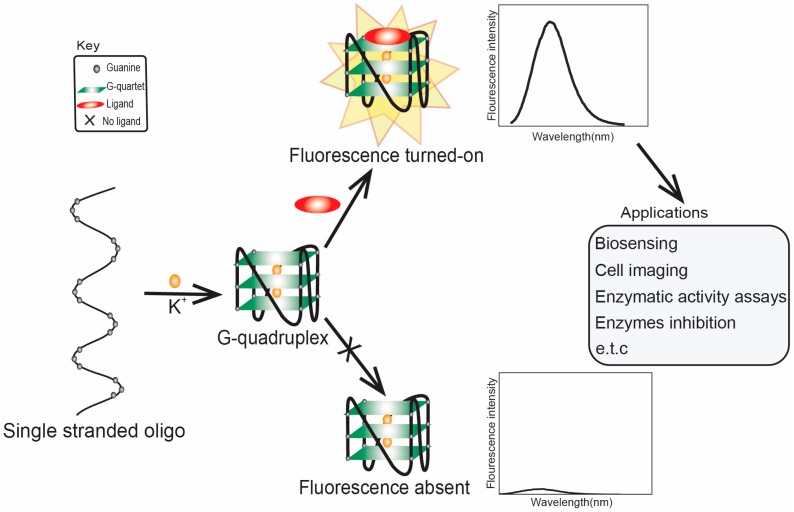
Schematic representation of ligand-enhanced fluorescence of G4. In the presence of ligand (top), it binds to G4 and results in enhancement in fluorescence. While in the absence of ligand (bottom), there is no such G4-ligand interaction, and hence no enhancement in fluorescence. This approach has been applied in different areas including but not limited to biosensing [72], cell imaging [51,52,73], enzymatic activity assay [74], and detecting G4 ligand inhibition of some enzymes [75,76] such as telomerase and ferrochelatase.

The biological significance of G4s in cells has led to a quest to develop diverse ligands that could help researchers understand their different cellular roles. On the one hand, some of these ligands are designed as fluorescent/imaging probes to verify G4 formation. On the other hand, some ligands can stabilize G4s and serve as chemical tools to challenge and alter G4-dependent processes. Also, it is possible that some ligands can perform both functions. In this review, we mainly focus on the organic G4 probes that is fluorogenic for in vitro and in cell detection of G4. As shown in Figure 2, these organic ligands in aqueous solvents have low fluorescence intensities; however, upon interacting with G4, an increased fluorescence intensity is observed, making fluorescence detection and imaging of G4 in vitro and in cells possible [51,52]. It should also be of note that there are also large repertoires of inorganic G4 probes that are luminogenic, and they have been extensively studied and several excellent reviews can be found elsewhere [53,54,55,56]. One representative example includes iridium (III) complex based G4s sensing methods; luminescent G4 switch-on probe for highly selective and tunable detection of cysteine and glutathione based on iridium (III) complex [57]. This method showed an enhanced intensity in the presence of desired metabolites [57]. Other examples of iridium (III) complex G4s based methods have also been reported, for instance, detection method for nicking endonuclease Nb.BsmI activity [58], for prostate specific antigen detection [59], thymine DNA glycosylase activity detection [60], for the detection of Siglec – 5 [61] and for ribonuclease H detection [62]. Platinum (II) complexes are another representative example of inorganic G4 ligand; Ma et al [63] reported the synthesis of platinum (II) complexes containing dipyridophenazine ligand as a highly sensitive luminescence probe for the detection of G4s and also showed to inhibit human telomerase enzyme (property also seen with organic ligands) and occur via an end stacking approach with a binding affinity of ∼10^7^ dm^3^ mol^−^^1^ [63]. Other examples of platinum (II) complexes reported includes the detection of nanomolar silver (I) ion in solution [64], as luminescence probe for G4 and c-myc downregulation [65]. Also, terpyridine ligand containing platinum (II) complexes have been shown by Sunthaaralingam et al. [66] to strongly binds to G4s of hTelo and c-myc through π-π stacking [66], which binding affinity and selectivity influenced by their aromatic surface [67]. Additionally, Ruthenium (II) complexes have been also reported as a selective luminescence probe for G4 detection, and occur via stacking of the ligand onto the G-tetrad and also based on insertion of the complex into the groove [68]. Other examples of ruthenium (II) complexes were also reported; for instance for sensing and methylation of duplex and G4s using Ruthenium (II) complexes containing dipyridylphenazine (dppz) ligand [69] for selective binding to various G4s using a bromo-substituent to the dipyridylphenazine [70]. Some advantages of inorganic fluorogenic ligands include their tunability, distinct properties (like anticancer drug development and their ability to induce G4s), and structures [66,68,71]. 

In the following, we will focus on the organic fluorogenic ligands/probes. Several representative classes and examples of each class are highlighted below:

### 2.1. Porphyrins 

Porphyrins exist in nature and are utilized by living organisms as co-factors in different enzymatic processes [77]. Ligands in this class inhibit telomerase via stacking with the G-quartet of G4 and its subsequent stabilization [78]. As shown in Table 1, *N*-methyl meso porphyrin IX (NMM) is an asymmetric anionic porphyrin and a major example of the porphyrin class of ligands. It has fluorescence excitation and emission wavelengths of 393 nm and 610 nm, respectively. It shows favorable binding to parallel G4s compared with anti-parallel G4s [79,80], and thus has the potential to discriminate between different strand orientations based on its fluorescence fold enhancement [81]. 

#### 2.1.1. Application of NMM and its Derivatives (TMPyP4 and TMPipEOPP)

NMM ligands have been applied in diverse applications, including enzyme activity and inhibition, cell imaging, and microbial detection, which are discussed below. 

The NMM inhibitory effect was demonstrated by Huber et al. [75], in which NMM was applied as an inhibitor of G4 unwinding by stabilizing and preventing helicase from accessing the desired G4 strand [75]. In 2010, Hu and coworkers [76] demonstrated a G4-based fluorescence assay that allowed both real-time monitoring and inhibition of RNase H. This method required an RNA–DNA substrate (with the DNA strand containing G4-forming sequences). In the presence of RNase H, the RNA strand gets cleaved and the DNA strand gets released, which then folds into G4 and subsequently binds with NMM and produces an enhanced fluorescence intensity [76]. Ren et al. [82] reported the use of NMM with tetrakis(diisopropylguanidino)-zinc-phthalocyanine (Zn-DIGP) to develop a dual fluorescent probe for the detection of nucleic acids. This approach was shown to be applicable with urines and serum samples [82]. NMM was also applied in a live-cell imaging study. When added to the cells, a large Stokes shift and a red-shift emission were observed, both of which were higher than the emissions seen with a different class of ligand, thioflavin T (ThT) [83]. This could be due to the green fluorescence emission of ThT, which can easily coincide with the intrinsic fluorescence of the cell’s other components [83]. 

Interestingly, NMM was also applied to microbial pathogen detection using integrated quaternized magnetic nanoparticles and a DNA amplification assay coupled with NMM. This method was based on the conformational transition from hairpin to G4 (assisted by Exo III nuclease) and subsequent specific interaction of the G4 with NMM. The method was able to detect as few as 50 cells mL^−1^ and 80 cells mL^−1^ of *E. coli* and *S. aureus*, respectively [84]. In 2016, Waller et al. [85] demonstrated ligand-specific regulation of nitrate assimilation in *Paracoccus denitrificans* (a Gram-negative soil bacterium). This method was based on stabilization of the *nasT* gene (which contains G4) by the 5,10,-15,20-tetra-(*N*-methyl-4-pyridyl)porphine (TMPyP4) ligand. Although NMM is an asymmetric porphyrin, cationic derivatives such as 5,10,15,20-tetra-4-[2-(1-methyl-1-piperidinyl)ethoxy]phenyl porphyrin (TMPipEOPP) and TMPyP4 were also applied as significant G4 binding ligands [86]. Notably, the TMPipEOPP ligand was shown to allow visual discrimination between G4s, duplexes and single-stranded DNA [86]. Limitation of some of these derivatives include off-target effects that lead to cell cytotoxicity [87] and they were not shown to have inhibitory properties like NMM [87]. More studies into these aspects are needed to fast track and improve the potentials of these ligands in live-cell investigations at both the macro- and micro-organism level. 

#### 2.1.2. Mechanism of NMM and its Derivatives (TMPyP4 and TMPipEOPP)

The mechanisms of interaction by this class of ligand were demonstrated to occur through both direct interaction with G4 and indirect interactions such as partial charge neutralization [88]. It was hypothesized that the interaction of porphyrin and G4s is based on intercalation with the adjacent G-quartets [77]. It was later shown that, when bound to G4, NMM fine-tuned its shape to fit the end face of the G4, resulting in enhanced fluorescence [76,89,90]. Another insight into the interactions was demonstrated in a triplet excited and decay study of zinc cationic porphyrin [5,10,15,20-tetrakis (1-methyl-4-pyridyl)-21H, 23H-porphine] (ZnTMPyP4). The interaction was demonstrated to occur via π–π stacking of the G4s ([AG3(T2AG3)3, (G4T4G4)2, and (TG4T)4]) and the macrocycle of ZnTMPyP4 [91]. The parent ligand, TMPyP4, from which ZnTMPyP4 was derived, was also shown to inhibit telomerase via external stacking on the G-tetrads [92]. However, the mechanism of interaction for the TMPipEOPP ligand was demonstrated to be dependent on the concentration of either the ligand or the targeted G4. At lower concentrations, one G4 binds two TMPipEOPP ligands via an ‘end stacking and outside binding’ approach [86]. Wheelhouse et al. previously demonstrated a similar effect [92]. At higher concentrations, two G4s bind one TMPipEOPP ligand via a ‘sandwich end-stacking’ approach [86].

### 2.2. Benzothiozole 

Thioflavin T (ThT), a 3,6-dimethyl-2-(4-dimethylaminophenyl) benzothiazolium cation, is also a commercially available dye like NMM, but unlike NMM, ThT is cationic (benzothiozole). ThT has excitation and emission wavelengths of 425 nm and 490 nm, respectively. It also has the advantage of low background fluorescence intensity, which translates to a high signal-to-noise ratio [6,87]. Prior to 2013, ThT was mainly used to bind other structures such as protein fibrils and amyloids through extensive π-stacking with tyrosine and tryptophan amino acids [93]. ThT was also demonstrated to inhibit interactions between fibrils and proteins [94]. 

#### 2.2.1. Application of ThT, its Derivatives (ThT-DB, ThT-HE, & ThT-NE), and IMT 

Because of its high sensitivity, ThT attracts the attention of chemists and has been applied in diverse applications, including biosensing, G4-specific probes, toxin detection, cell imaging, and microbial detection, among others, which are discussed below. 

ThT was first reported in 2013 as a G4 ligand to study the human telomere G4 22AG [dAGGG(TTAGGG)3], and it was demonstrated to differentiate between G4, duplexes and single strands with high fluorescence intensity [87,95]. This fluorescence turn-on ligand has been widely applied as a sensor, for instance, for Ag^+^ [96] and Hg^+^ [97] detection, based on the interaction between the ligand and G4. ThT has also been applied as a label-free fluorescent turn-on ligand for sensing bio-thiols based on its ability to induce unique G4 structures [72], and it was demonstrated as a probing method for structural changes in i-motif (four stranded DNA secondary structures that consist of hemi-protonated and intercalated cytosine base pairs (C:C^+^)) [98]. It was applied as a highly sensitive sensor for thrombin detection using Förster resonance energy transfer (FRET). This method is based on the ability of ThT to induce G4, which is then used as an energy acceptor, with a conjugated polymer on the other side as the energy donor [99]. More recently, ThT was applied as a G4-based aptasensor for the detection of adenosine deaminase activity and inhibition [74]. ThT was also applied in toxin detection, as demonstrated using a G4-based aptasensor that selectively quantified the amount of toxins in food materials. This method was based on an aptamer (selected against a toxin) binding to ThT to form a G4–ThT complex (in the absence of the target toxin). When the toxin is present, it binds to the G4-based aptamer, which leads to the release of ThT, and a subsequent change in fluorescence is observed [100]. As shown in Table 1, unlike NMM, some G4 studies have indicated that ThT-induced G4s can potentially cause topological changes [101], producing false positive and false negative results [97]. It was also shown to bind tightly to non-G4 G–A-rich containing sequences and dimerise them into a parallel double-stranded modes [96]. Furthermore, ThT was found to be difficult to use for effective monitoring of G4s in the chromatin of live cells because of its inability to stain the nuclei [102]. This led to the synthesis of some ThT derivatives. 

Some derivatives of ThT have been reported, such as ethyl-substituted ThT, which was applied as a fluorescence probe with high specificity for G4 structure detection and discrimination from other nucleic acid forms [103]. Interestingly, this method allows naked-eye visualization of G4 in solution under ultraviolet light. In a similar study, Kataoka and coworkers [101] synthesized two derivatives of ThT by replacing the N3 methyl on the benzothiozole ring with either a ((*p*-(dimethylamino)-benzoyl)-oxy)-ethyl group (ThT-DB) or a hydroxyethyl group (ThT-HE) and applied them as parallel G4 probes. Their results showed over 200-fold enhancement in fluorescence intensity compared with normal ThT, and also great specificity to parallel G4s. Other benzothiazoles have been reported, such as IMT, which can selectively bind G4s in a cell’s chromatin (with negligible cytotoxicity). It can be applied in vivo to demonstrate the changing response of G4s to different chemicals in real time [51]. This method is simpler than the triangulenium method reported earlier [52], which requires a longer acquisition time and specialized equipment. 

Lastly, ThT has also been applied in studies of G4 prevalence in micro-organisms. For example, in 2016, Burrows and coworkers [104] applied ThT as a fluorescence probe to study the prevalence of G4s in the zika virus. Two years later, the same group applied ThT as a fluorescence probe for the detection of G4s in *Chlamydomonas reinhardtii* [105]. That same year, Zahin et al. [106] applied ThT for the identification of G4-forming sequences in papillomaviruses (using ThT as a fluorescence probe to screen for G4-forming sequences). Similarly, ThT derivatives have been applied in viral RNA genome detection and monitoring. This was demonstrated very recently by Luo et al. [107], who developed the ligand ThT–NE, with the excitation and emission wavelengths shown in Table 1 (ThT derivative). The ligand was a cell permeable and highly specific G4-based fluorescence turn-on probe for real-time imaging of native viral RNA in the hepatitis C virus (HCV). This method was shown to allow subcellular monitoring and continuous live-cell monitoring of infected cells [107]. However, the limitation of this ligand class include the fact that only few were shown to penetrate the cells [107] and reach their desired target. The possible reasons could be due to their physical size, non-selectivity in complex samples or conditions or the potential to form aggregates in cells [108]. Some of the other imperative factors in designing novel fluorescence G4 probes include permeability, affinity, selectivity, and cytotoxicity. Some G4-containing aptamers (such as Mango) have been shown to discriminate between this class of ligands via a concerted mechanism, whereas others (such as Spinach) enhance the fluorescence intensities of many ligands with no discriminating properties between them [109]. Hence, there is a need for G4 ligand with higher specificity, affinity, and low toxicity for live cell application. 

#### 2.2.2. Mechanism of ThT, ThT-NE, and IMT

The mechanism of interaction between ThT and G4s was demonstrated to be ligand concentration dependent, in which several ThT ligands bound cooperatively to the 5′-G4 unit [87]. Unlike the NMM derivative TMPipEOPP (which also depends on ligand concentration), in this case, the fluorescence enhancement was higher when a single ThT ligand was bound to G4. The enhanced fluorescence intensity was demonstrated to be a result of the restriction in circular movement and subsequent conformational changes between the benzothiazole and dimethylaminobenzene rings [87]. However, the fluorescence intensity diminished when more than one ThT ligand was bound to the rearranged/changed G4 structure [87]. It was also demonstrated that the interaction between ThT and G4 may be due to end stacking with the upper G-tetrad of RNA G4; that is, the benzothiazole unit stacks onto the upper G-quartet of the G4, thereby donating most of the π-stacking force in its binding [110]. Similarly, the mechanism of interaction for ThT-NE was demonstrated to occur via pi–pi stacking of the ligand and the ending G-quartet of the G4, resulting in rotational restriction of the ligand. Likewise, the mechanism of IMT interaction with G4s was shown to occur via stacking to the terminal (5′-end) G-quartet [51].

### 2.3. Triphenylmethane (TPM)

The TPM class of ligands has many members, including methyl violet (MV), ethyl violet (EV), methyl green (MEG), malachite green (MG), and crystal violet (CV). This class was shown to distinguish intramolecular from intermolecular G4s and single DNA strands from duplex DNAs [111]. For this review, we focus on CV and MG. Prior to its application in G4 detection, CV was widely used as a dye for staining papers, textiles, drugs, and food materials [112]. It then began to attract enormous attention as a stain for biological studies. It has fluorescence excitation and emission wavelengths of 540 nm and 640 nm, respectively, as shown in Table 1. 

#### 2.3.1. Application of CV and MG 

Like NMM and ThT, CV has been widely applied in diverse areas, including sensing that can distinguish single strand and duplex structures from G4 [113,114,115], and it preferentially binds to intramolecular rather than intermolecular G4 [113]. It is also applied in biosensing and thrombin detection. Other applications are discussed below.

G4-based aptasensors (discussed in detail in Section 3 of this review) are attracting enormous scientific interest. Nonetheless, in this section, we touch on the G4 ligand-based fluorescence turn-on aspect of some representative aptasensors. One example is an aptasensor selected through modified-affinity chromatography to replace G4 binding [116] and subsequent detection of CV. CV was also applied as a biosensor for the detection of Pb^2+^ based on the electrochemical current of a G4–CV complex [114]. He et al. [117] developed a label-free G4-based aptamer probe for the selective detection of ATP in aqueous solution using CV as a G4 fluorescent probe. In this method, the ATP aptamer is in a duplex format (i.e., hybridized to its complementary sequence); in the absence of ATP, it gives a weak fluorescence intensity. However, in the presence of ATP, the duplex dissociates, resulting in an aptamer–G4 complex via a ‘population shift mechanism’. The presence of CV results in its specific binding to the G4 complex, thus enhancing the fluorescence (depicted in Figure 2). CV was demonstrated to distinguish between parallel and anti-parallel topologies [118]; it preferentially binds to anti-parallel G4 and produces enhanced fluorescence intensity due to the shielding effect of the G4 end-loop on CV against the solvent, whereas the parallel G4 cannot provide CV with such a shield due to the lack of the end-loop. 

Jin et al. [119] reported another G4-based aptasensor and demonstrated its ability to detect human thrombin protein. This method was based on the enhanced fluorescence of CV as a result of its binding to a thrombin–G4 aptamer complex. In 2009, Kong et al. [113] demonstrated a simple and sensitive method for discriminating between G4s, single strands and duplexes based on the fluorescence enhancement of CV or CV energy transfer fluorescence. Interestingly, in the presence of C-rich sequences (complementary strands to G-rich), this method was shown to measure the amount of G-rich sequences that partake in G4 formation based on the fluorescence enrichment of G4–CV complexes. That same year, a novel biosensor for the homogenous sensing of K^+^ was also reported. This biosensor was based on increasing and decreasing fluorescence intensity with increasing K^+^ [120]. A similar approach (of decreased fluorescence with increasing K^+^) for the determination of K^+^ was reported. However, this method was based on the interaction between G4 containing a thrombin-binding aptamer (TBA) and CV. The interaction of TBA with CV (in the absence K^+^) produces enhanced fluorescence. However, in the presence of K^+^, TBA-based G4 is formed, and when it interacts with CV, the difference in fluorescence intensity is measured (depending on the K^+^ concentration) [121]. Thus, the amount of K^+^ can be determined. MG has also been applied as fluorescence G4-based aptasensors for binding recognition to MG ligands. However, the limitation of this class includes the fact that it does not allow naked-eye visualization of G4s in solution. Also, they have different binding modes such as the stacking and end loop protection modes (as discussed in Section 2.3.2 below), and G4 and other nucleic acid structural motifs and topologies can sometimes significantly influence the ligand’s fluorescence enhancement [122].

#### 2.3.2. Mechanism of CV and MG

In 2009, the mechanism of interaction was demonstrated to occur via stacking of CV to the two outside G-quartets of G4 [118] and the binding of two CVs per one G4. This stacking increased the rigidity of the ligand and subsequently the fluorescence intensity. In the same year, Kong et al. demonstrated the end-loop protection mechanism of bound ligands in an antiparallel topology [120]. The stacking mechanism was also reported for CV interactions with i-motif [123]. 

### 2.4. Other Ligands Reported in the Literature as G4 Fluorescence Turn-On Ligands

As summarized in Table 1, several fluorescence turn-on ligands were also demonstrated to recognize G4. However, acridine-based ligands are largely used as efficient G4 stabilizers, such as trisubstituted acridines. *N*,*N*′-[9-[[4-(Dimethylamino)phenyl]amino]-3,6-acridinediyl]bis[1-pyrrolidinepropanamide], known as BRACO-19, has attracted much scientific interest due to its G4 stabilization and inhibition of telomerase enzyme activity [37,124,125]. It also shows antiviral activity as it impairs HIV-1 long terminal repeats promoter activity, which controls the viral gene transcription [126]. Pyridostatin (PDS) was also reported to bind to G4 with high specificity through an end-stacking approach [127,128,129], but it did not fluoresce. Later they synthesized a PDS analogue which allows the evaluation of the cellular localization of the drug (“by promoting telomere dysfunction and long-term growth inhibition in human cancer cells”) [130], more so, they explored how PDS interferes with the roles of proteins that operates on G4s and how that in turn affects targeting of G4s by small molecules [131]. This changed with the very first fluorogenic acridine dyes containing cyanine, which allowed a wide spectrum ranging from orange to the near infrared region, as demonstrated by Mahmood and coworkers [132]. Later on, the same group (motivated again by the incredible potential of BRACO-19) demonstrated the development of a tri-substituted (3,6,9-trisubstituted acridine; cyanine dye 1) water-soluble acridine-based dual probe, a pH-sensitive and G4 fluorescence probe containing monomethine cyanine dye (which has fluorescence excitation and emission wavelengths of 400 nm and 475 nm, respectively) [133]. Cellular pH is an essential factor in cell activities, and the probe was demonstrated to be sensitive to a pH range of 5–9. In acidic conditions, the probe showed enhanced fluorescence due to the protonation of acridine. A positive charge delocalises between the acridine and indole moieties and fluorescence is reduced at higher pH values (as the acridine can no longer be protonated). The system was reported to operate based on a ‘push–pull mechanism’ [133]. The limitation of this ligand is that its application was not demonstrated in vivo. BRACO-19 was shown to bind to G4 via three modes of interaction: stacking to the top quartet, intercalation on the lower quartet and groove binding [124,134]. 

Other reports on G4 fluorescence turn-on ligands include that of Jin et al. [135]. In 2014, they applied a BPBC ligand composed of benzimidazole and carbazole groups as a fluorescence turn-on probe for parallel G4 detection. The ligand was shown to bind parallel G4s via an end-stacking approach. It was also shown to have incredible selectivity towards parallel G4s due to its possession of a ‘crescent-shaped pi-conjugated planar core’, which is bigger than the G4 plane dimension. Likewise, Yang et al. [136] reported a new class of bis(4-aminobenzylidene)acetone derivative called GD3 as an effective red-emitting fluorescence turn-on ligand for parallel G4s. They demonstrated the biological application of this ligand in fixed cells and showed that it allows the visualization and monitoring of G4 structures. The mechanism of interaction was the dipole moment created in the microenvironment of the ligand and the restriction of the fluorophores, resulting in altered charge transfer in the system and hence enhanced fluorescence [136]. However, the limitation of this ligand is that it can only allow monitoring of G4s in fixed cells. Other parallel G4 binding ligands were reported by Chen and coworkers [137], who demonstrated the use of 2,4,5-triaryl-substituted imidazole (IZCM-1) as an effective ligand that binds specifically to parallel G4s without affecting their topology or thermal stability. Later, the same team [138] synthesized another G4 fluorescence turn-on triaryl-substituted imidazole ligand called [2-(4-(4,5-bis(4-(4-methylpiperazin-1-yl)phenyl)-1*H*-imidazol-2-yl)phenyl)-6-(4-methylpiperazin-1-yl)-1*H*-benzo[de]isoquinoline-1,3(2*H*)-dione] (IZNP-1) and demonstrated its application to highly and specifically target telomeric multimeric G4 structures (i.e., it can discriminate between telomeric multimeric G4s and monomeric G4s) through intercalation of the ligand into the ‘pocket’ of two G-quartet units of G4. This ligand was demonstrated to induce apoptosis and senescence in cancer cells as result of telomeric DNA damage and telomere functional disruption due to the ligand intercalation into the G4 structure [138]. Shavalingam et al. [52] applied a triaryl methyl carbocation (triangulenium) derivative called DAOTA-M2 that localizes in the nuclei (with low toxicity) and interact with G4. Also, a “one-to-one G4-specific sensor”, IZFL-2, that can distinguish between different G4s was demonstrated [139]. This method allows the visualization of interactions between ligands and G4s by fluorescence lifetime microscopy. The binding mechanism of this ligand occurred via π–π stacking between the guanine moieties of the outer G-quartet and core of the ligand [140]. 

Most of the fluorescence turn-on probes can only accommodate one output. In 2014, Yan et al. [141] developed a multifunctional probe called (*E*)-3-((7-(diethylamino)-2-oxo-2*H*-chromen-3-yl)methylene)-6,7-difluoro-4-methyl-9-oxo-1,2,3,9-tetrahydropyrrolo [2,1-*b*]quinazolin-4-ium iodide (ISCH-1) that utilized two different outputs (i.e., colorimetric and fluorescence). These types of probes are reliable and applicable to diverse applications. The ligand was designed based on an isaindigotone framework that incorporated coumarin–hemicyanine to achieve a multifunctional probe. The application of this probe to detect G4s was demonstrated [141]. The limitation of this ligand is that it cannot allow specific targeting of G4s at a given RNA region (such as the 5′ UTR). To address this issue, Chen et al., refined ISCH-1 by attaching an oligonucleotide (which had a complementary sequence to an adjacent sequence of the G4 sequence of interest) that would allow subsequent fluorescence in situ hybridization (FISH) to be performed. Hence, the probe consisted of two distinct segments, the fluorescence turn-on and oligonucleotide hybridization segments. They referred to the probe as a G4-triggered fluorogenic hybridization (GTFH) probe [142]. The refined ISCH-1 ligand was called (*E*)-3-((7-(diethylamino)-2-oxo-2*H*-chromen-3-yl)methylene)-7-fluoro-4-methyl-9-oxo-6-(prop-2-yn-1-yloxy)-1,2,3,9-tetrahydropyrrolo[2,1-*b*]quinazolin-4-ium (ISCH-oa1) [142]. The application of this ligand was demonstrated with 5′ UTR of *NRAS* mRNA by incorporating an oligonucleotide complimentary to the adjacent sequence of the *NRAS* G4 sequence to form ISCH-nras1 ligand that can selectively bind and uniquely allow the visualization of G4s in this region both in vitro and in cells [142], however, this ligand has limitations of not able to detect the ‘in-situ spots’ of a given RNA in single cell and also requires RNAs to be transfected into cells to increase their concentration. Amazingly, the same team developed yet another ligand that was also based on an isaindigoton framework, but it contained coumarin aldehyde and an *N*-methylated quinoline moiety, this ligand was named (*E*)-2-(2-(7-(diethylamino)-2-oxo-2*H*-chromen-3-yl)vinyl)-6-fluoro-1-methyl-7-(4-methylpiperazin-1-yl)quinolin-1-ium iodide (QUMA-1). Unlike GD3 ligand that was only shown in fixed cells, QUMA-1 was demonstrated through live-cell imaging to be a highly selective fluorescence turn-on probe for real-time and continuous tracking and monitoring of rG4 structural dynamics in live cells. It was also applied in the visualization of rG4s unwinding by helicase [143]. Nonetheless, the fluorescence intensity of this ligand decreases in the presence of other competing G4s ligands. The interaction between QUMA-1 and rG4 was demonstrated to be caused by the rotational constraint experienced by the ligand at higher energy levels because of a conformational rearrangement [143].

Laguerre et al. [144] reported another multifunctional G4 smart probe (ligand and fluorescence turn-on probe) developed using the template-assembled synthetic G-quartets (TASQ) method. They used TASQ to develop pyrene template-assembled synthetic G-quartets (PyroTASQ) as both a smart G4 ligand and a fluorescence probe. This ligand and probe were demonstrated to recognize and bind to both DNA and RNA G4s and it was shown to occur through an interesting approach, in which the ligand causes a ‘quadruplex-promoted conformational switch’ that leads to the assembling of four guanines into a G-quartet. Subsequently, the pyrene’s fluorescence is released [144]. However, the application of PyroTASQ to detect G4s in live cells proved difficult as it aggregates in the cells [108]. To address this issue, the same group demonstrated another multitasking G4 probe synthesized in an approach similar to that of PyroTASQ but replacing the pyrene group with naphthalene to form a Naptho-TASQ (N-TASQ) [108]. The authors were able to visualize RNA G4s in live cells using the multi-photon microscopy method [108] and both RNA and DNA imaging using confocal microscopy [145]. The interaction occurs through an approach similar to that of PyroTASQ [108]. However, no binding competition with other G4 ligands was shown.

### 2.5. Future Perspectives of the Development and Applications of Fluorescent Turn-On Ligands

As seen from above sections, most ligands have different binding modes, and G4 and other nucleic acid structural motifs can sometimes significantly influence the ligand’s fluorescence enhancement [122]. This raises some concerns that need to be addressed: is the ligand-binding mechanism to G4s dependent on the loop length, bulge, inter versus intra-molecular G4s, parallel versus antiparallel G4 topologies, or number of G-quartets? Understanding the binding modes between the ligands and G4s, as well as the dimensions of the G4s grooves, is critical to understanding the mechanism of interactions between the ligands and G4s. Also, future high-resolution 3D structures with bound ligands will potentially allow a better picture of the ligand binding modes for future ligand design and G4 targeting.

Also, some of these ligands showed a decreased fluorescent enhancement in presence of other competing ligands, as such, ligand competition studies with other ligands are needed to fully ascertain the selectivity of the ligands of interest, this will allow the design and development of ligands with better fluorescent properties and this could prevent the off-target effects of some of these ligands (that leads to cell cytotoxicity) [87], and can also address the false positive and false negative results [97] produced by some of the ligands as a result of their abilities to induce topological changes [101]. More so, as mentioned earlier, only few of these ligands can effectively penetrate the cells [107] and reach their desired target [108]. Therefore, permeability, affinity, and selectivity are critical factors that need to be further improved when designing and developing advance novel fluorescence G4 probes.

Lastly, application-wise, many of these ligands are not yet applied in vivo [133] and can only allow monitoring of G4s in fixed cells [136], while others still requires RNAs to be transfected into cells to increase their concentration and thus signal [142]. Therefore, more advanced ligands are required with an improved property to reach their targets in cells and across different species, as well as increase their potential for real-time monitoring and single G4 detection application. We anticipate that, by addressing these issues, we could shed lights into the better understanding of the folding status, dynamics, and localization of G4s in cells and their biological roles in different cellular processes.

## 3. G-Quadruplex-Containing Nucleic Acid Aptamers

While biologically-relevant G4 targets can be detected and visualized by fluorescent turn-on ligands in vitro and in cells as described above, another exciting and emerging field of research is the identification and development of G4-containing aptamers, which may serve as molecular tools for diverse chemical and biological applications. Aptamers are single-stranded DNA or RNA sequences that are able to recognize natural and synthetic targets ranging from metal ions, small molecules, dyes, proteins, toxins, microbes, and cells [149]. The most well-established screening method for nucleic acid aptamers is the iterative SELEX process, which selects aptamers for targets of interest from a library of random sequences [150]. Aptamers are proposed to function as alternatives to other affinity reagents (e.g., protein-based antibodies) owing to several key advantages, such as simple synthesis and modification, design flexibility, high target specificity, and good stability. These properties can be successfully exploited in drug delivery, molecular imaging, clinical diagnosis, and biochemical research [151,152].

Aptamers can adopt various structural arrangements, which enable their recognition functionality. Among several architectures, the G4 structures are commonly found in aptamers [153]. One possible reason is that G4s involve sophisticated tertiary folding and display remarkable structure polymorphism and tunable conformation depending on the oligonucleotide sequences and different conditions of cations, ligands, or pH level [48,154], which give them strong structural discriminatory ability and contribute to their affinity and specificity for target binding. In addition, the high negative charge density of G4 gives them an advantage in the selection process for binding to positively charged surfaces of targets, such as proteins with positively charged amino acids (e.g., arginine, lysine, histidine) and metal ions via electrostatic interactions [153]. The first reported G4-containing aptamer was a 15-nucleotide thrombin-binding DNA aptamer, with the sequence of d(GGTTGGTGTGGTTGG) (Table 2), first selected using SELEX to bind thrombin [155]. The aptamer structure was later determined in NMR study to consist of two G-quartets [156]. Other G4-containing DNA aptamers have since been reported against targets including proteins and small molecules (Table 2). RNA is also equipped to form aptamers owing to its great structural flexibility that recognize small molecules, as exemplified in naturally occurring riboswitches [157,158]. A list of representative G4s-containing DNA and RNA aptamers is shown in Table 2.

**Figure 3:**
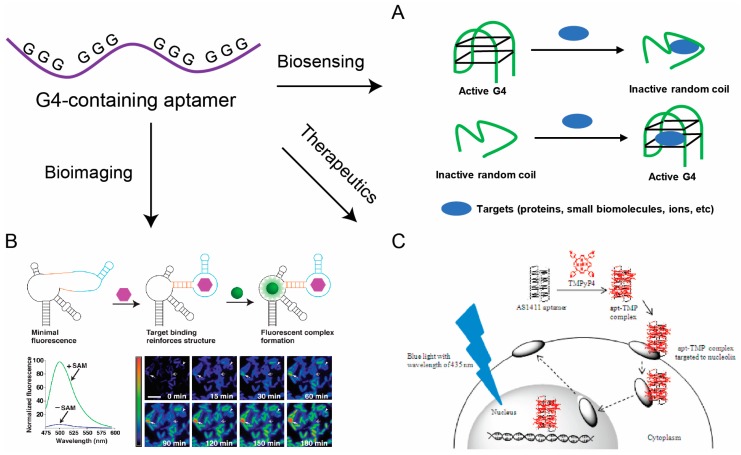
Representative applications of G-quadruplex-containing aptamers in biosensing, bioimaging and therapeutics. (**A**) Biosensors based on the conformational change of G-quadruplex-containing aptamers. Targets binding can destabilize/stabilize the G4 structure of aptamers and this conformational change is designed to cause signal change in the system. (**B**) Imaging metabolite (e.g., SAM) in living cells with fluorogenic RNA [159]. Reprinted with permission from [159]. Copyright 2012 American Association for the Advancement of Science. (**C**) Proposed mechanism of a photodynamic therapy strategy by using AS1411 as drug carrier to target cancer cells [160]. Reprinted with permission from [160].Copyright 2010 American Chemical Society.

The existence of G4 structures in aptamers ideally combines the superior properties of G4 and the intrinsic binding capabilities of aptamers. These features favor the application of G4-containing aptamers in biosensing, bioimaging, and therapeutics, as described below (Figure 3).

### 3.1. Biosensing Applications of G-Quadruplex-Containing Aptamers

Biomolecules are of great importance in regulating various biochemical reactions and cellular metabolic processes. Aptamer-based biosensors have been widely used for biomolecule detection and for understanding their biological functions [151,161]. In addition, by using disease-related biomolecules as analytes, aptamer-based biosensors have become powerful diagnostic tools [162]. Various sensing strategies and signal readout techniques are used to develop sensing systems for a variety of targets [151]. Here, we summarize the fluorometric biomolecule aptasensors that utilize the G4 structure of aptamers in strategic design. Most aptasensors are based on the conformational switching of aptamers. Target binding can cause distortion on aptamers and stabilize or destabilize the G4 structure (Figure 3A). This conformational switch can be effectively monitored by several fluorescence signal output methods, such as nanomaterials [163,164], molecular beacons [165] and organic dyes, especially G4-selective fluorogenic ligands [99,117,166,167,168,169], which can give a fluorescence response upon target binding. Examples of the use of fluorometric G4 aptamer-based biosensors include the detection of proteins, small biomolecules and cations.

Regarding protein detection, thrombin has been employed as an analyte in some aptasensors. Li et al. [163] developed a FRET aptasensor for thrombin in both buffer and blood serum based on a fluorescein amidite (FAM)-labelled aptamer and graphene. A single-stranded thrombin aptamer was absorbed onto the surface of graphene due to noncovalent assembly and the fluorescence of FAM was quenched because of the FRET effect. With the addition of thrombin, it interacted with the aptamer and formed a G4-thrombin complex, which had weak affinity to the graphene and thus dissociated from the graphene, resulting in fluorescence recovery. A similar aptasensor was developed by Chu and coworkers using MoS_2_ nanosheets and an FAM-labelled thrombin aptamer [164]. In some other assays, nucleic acid-interacting dyes are used to avoid labelling of aptamers. Zhou et al. [166] designed a thrombin aptasensor based on a four-branched pyrazine derivative (TASPI). The thrombin aptamer can eliminate the fluorescence of TASPI, whereas in the presence of thrombin, its aptamer specifically bound to thrombin and folded into a G4 structure, releasing TASPI molecules. Liu et al. [99] reported a FRET-based aptasensor for thrombin by using ThT as an energy acceptor and a water-soluble conjugated polymer (CP) as an energy donor. In this approach, ThT was bound to a thrombin aptamer (TBA) first, which induced TBA to fold into a G4 structure, forming a fluorescent ThT–TBA complex. The electrostatic attractions between the ThT–TBA complex and CP resulted in a high FRET signal. While in the presence of thrombin, TBA formed a G4-thrombin complex first, resulting in a longer distance between ThT and CP, which led to a low FRET signal. This method can also be used for human serum thrombin detection.

Regarding G4 aptamer-based fluorometric biosensors for small biomolecules, ATP has been used as a target in many assays because of its biological significance. In these assays, G4-selective fluorescent ligands were widely used to transduce the target binding into a fluorescence signal change. For example, Ji et al. [167] developed an ATP detection method using an ATP aptamer and ThT. The G4 structure of the ATP aptamer allowed the intercalation of ThT to produce strong fluorescence. However, upon ATP binding to its aptamer, a conformation change occurred in the aptamer. ThT was released into the solution, causing drastic suppression of the fluorescence intensity. This method was capable of detecting ATP in human serum and cell extracts. Other methods adopting similar principles have also been reported for ATP detection by using other G4 selective fluorescent ligands, such as CV [117], zinc(II)-protoporphyrin IX [168], and berberine [169]. An alternative approach to detecting ATP was based on a molecular beacon as the signal output. Willner et al. [165] assembled ATP aptamers into hairpin DNA, which was modified with a fluorescent dye (FAM) as a fluorophore at its 5′ terminus and a black hole quencher (BHQ1) at its 3′ terminus. In the absence of ATP, FAM, and BHQ1 were in close proximity, resulting in fluorescence quenching of FAM due to the FRET effect. However, in the presence of ATP, the hairpin DNA switched to a G4 structure and was bound to ATP. The re-organized G4 hairpin structure allowed Exo III to hydrolytically digest the 3′-end strand, and thus BHQ1 was released into solution and the fluorescence of FAM was recovered.

Pei et al. [170] used G4 aptamer-based fluorometric biosensors for cation detection. They proposed a sensing strategy for Pb^2+^ based on target-induced G4 formation and found a G-rich sequence (AGRO100) that works as a Pb^2+^ aptamer and forms a G4 conformation induced by Pb^2+^. The G4-Pb^2+^ complex binds to NMM, giving a turn-on fluorescence response to Pb^2+^. Another interesting work reported by the Wei group achieved the in vivo detection of K^+^ in living organisms (brains and tumors) [171]. They selected a G-rich DNA probe that was selectively induced to form a parallel G4 by K^+^. The G4-K^+^ complex can enhance the fluorescence of PPIX. Thus, the concentration of K^+^ could be detected by modulating the fluorescence of the system. A similar study was reported by Tan et al. [172] for human blood K^+^ detection using a G4 aptamer of K^+^ and a G4-binding ligand (EBMVC-B). This was the first attempt to exploit G4 aptamer-based fluorescent sensing for direct assay of blood targets. This concept also holds great potential for other ions’ detection by selecting their corresponding G4-containing aptamers.

### 3.2. Bioimaging Applications of G-Quadruplex-Containing Aptamers

Monitoring the distribution and tracking of intracellular biomolecules contributes to the understanding of their cellular location, dynamics, and functions, which is vital for gene regulation, disease diagnosis, and drug discovery [173]. Fluorescence imaging is a major technique for identifying the expression and spatial and temporal dynamics of biomolecules [174]. A few G4-containing aptamer-based strategies have been reported for bioimaging applications.

A particular example of this application is RNA Spinach, which is a 98nt SELEX-identified RNA aptamer that can fold into a G4 structure and switch on the fluorescence of 3,5-difluoro-4-hydroxybenzylidene imidazolinone (DFHBI) [175]. RNA Spinach has been demonstrated to be a powerful bioimaging tool because of its strong resistance to photo-bleaching [175]. Spinach was successfully implemented for RNA imaging in living mammalian cells [175] and bacteria [176], as well for cellular metabolite [159] and protein [177] imaging in bacteria. The common strategy of Spinach-based bioimaging is to express ‘fusion RNAs’ that comprise Spinach and an additional RNA tag that can recognize targets and give a fluorescence response. For example, Jaffrey and coworkers [159] established a strategy to image cellular metabolites in *E. coli* based on Spinach (Figure 3B). They fused the target-binding aptamer to RNA Spinach via a transducer stem and destroyed the G4 motif of Spinach. Target binding to the aptamer promoted stabilization of the transducer stem, enabling Spinach to fold and activating DFHBI fluorescence. They also adapted this approach to monitor protein levels in *E. coli* [177]. In addition to the original Spinach, other versions of Spinach, like Spinach-mini [175], Spinach1.2 [178], Spinach2 [178], Spinach2-mini [179] and Baby Spinach [180], all adopt a G4 structure. A compelling alternative to RNA Spinach has been identified by in vitro selection, termed as Mango. Mango has a more rigid G4 structure, which activates the fluorescence of thiazole orange derivatives [109]. RNA Mango has also been used to bioimaging systems. For example, Jepsen et al. [181] developed a FRET sensing system by using RNA origami scaffolds consisting of Spinach and Mango. The fluorescent aptamers Spinach and Mango were placed in close proximity to obtain FRET and a new fluorophore was synthesized to increase the spectral overlap. The FRET-based constructs were finally expressed in *E. coli*. These bioimaging applications reveal the fact that G4-containing aptamers can provide promising platform for efficient intracellular monitoring of biomolecules in living cells and organisms.

### 3.3. Therapeutic Applications of G-Quadruplex-Containing Aptamers

For therapeutic functions, several G-quartet-containing oligonucleotides have been demonstrated to have potential as drugs, such as HIV inhibitors [182,183]. DNA sequences (termed 93del and 112del) adopting the G4 folding topology have been reported to exhibit anti-HIV1 integrase activity in a nanomolar range [182,183]. Another DNA aptamer (T30695) with a sequence similar to that of 93del and 112del but with a rather different G4 folding topology has also been identified as an HIV1 integrase inhibitor [182]. Despite the existence of quite a few anti-HIV1 integrase aptamers, delivering them to intracellular targets is still a challenge. Jing et al. [184] developed a system to deliver an HIV1 integrase inhibitor (T40214) into the target cell nuclei, which successfully decreased HIV1 replication, thus demonstrating the possible use of G4-containing nucleic acid aptamers as anti-HIV drugs.

The AS1411 aptamer is a 26nt G-rich DNA sequence that can fold into a G4 structures and bind to nucleolin with strong affinity and specificity [185]. AS1411 has been widely employed to target higher nucleolin-expressing cancer cells. For example, Shieh et al. [160] developed an aptamer-based photodynamic therapy strategy by using AS1411 as a drug carrier to target cancer cells (Figure 3C). Willner and co-workers [186] also designed a drug delivery method based on AS1411-functionalized metal–organic framework nanoparticles loaded with anti-cancer drugs. AS141 was used to target the cancer cells, and VEGF in the target cells can trigger the release of the anti-cancer drug. This concept could be adapted to other diseases that involve cellular biomarkers and their aptamers as gating units.

### 3.4. Current Challenges and Future Perspectives of the Development and Applications of G4-Containing Aptamers

Since the thrombin-binding aptamer (TBA, Table 2) was first identified as a G4-containing aptamer, G4 structure has been reported in a number of DNA and RNA aptamers towards various targets (Table 2). The potential applications of these G4-containing aptamers have been illustrated in different areas as discussed earlier. Nonetheless, the development and applications are still in the early stages and suffer from several challenges.

Firstly, high-resolution structures of targets with and without aptamer bound are necessary to reveal the structural basis of these complexes, which will allow development and optimization of aptamer’s sequence and structure, and provide insights to further enhance the aptamer’s properties for desired applications. So far, only a few G4-containing aptamers have been structurally determined (TBA, Spinach, Mango, etc.) [156,187,188], whereas the structures of other G4-containing aptamers are still poorly understood, making it challenging to improve the design and properties of those G4-containing aptamers. In addition, as the number of G4-containing aptamer is still limited, identifying novel G4-containing aptamers against new targets will broaden the scope of this research area.

Secondly, the folding of G4-containing aptamers should be experimentally investigated to ensure their specificity both in vitro and vivo, as the aptamer structure might re-fold in cellular environment. The selected aptamers’ folding can be inhibited by cell machinery and physiochemical environment when used in cells/in vivo, which decrease the aptamer’s ability to bind to targets. Therefore, more tests in different conditions are required to ensure the aptamer specificity. In addition, new experimental structure mapping techniques [32,48] and cell imaging have been developed to examine G4 folding, allowing us to verify the formation of G4 structure in the G4-containing aptamer in both in vitro and in vivo settings.

Thirdly, most of the G4-containing aptamer-based systems are still proof-of-concept studies, which were performed in vitro or in cells. The application of them from bench to diagnosis and therapy are still elusive and need to be fully investigated. One main obstacle for therapeutic applications is the biological barrier existing during the drug delivery process [161], such as cell membrane internalization. In addition, the nucleases present in biological system also pose another key issue. To solve these, more efforts should be made to develop G4-containing aptamer-based systems that can penetrate across the biological barrier, such as the use of other biocompatible species like nanomaterials to facilitate intracellular delivery of the therapeutic G4-containing aptamers [189]. To resist nuclease digestion and increase G4-containing aptamer’s lifetime in cells/in vivo, unnatural nucleotide base modifications can be used, such as 2′O-methylation, lock nucleic acids, and phosphothioate. Further improvement in this area will facilitate the better G4-containing aptamer stability and delivery in complex system. Taken together, with these challenges to be addressed, G4-containing aptamers will likely achieve their diagnostic and therapeutic potential, leading to a new chapter in the application of G4-containing aptamer research.

## 4. Conclusions

Over the years, impressive progress has been made in the G4 field with respect to G4 fluorescent turn-on ligands and G4-containing aptamers. Remarkable discoveries and applications have been reported in these two promising fields, including the interaction mechanism and applications of several classes of G4 fluorescent turn-on ligands (Table 1), as well as the G4-containing aptamers (Table 2) and their uses in biosensing, bioimaging, and therapy. With such developments achieved in the investigation and applications of G4s, the study of G4 fluorescent turn-on ligands and G4-containing aptamers is expected to open new perspectives towards wider biological understanding and applications of G4s both in vitro and in cells.

## Figures and Tables

**Table 1:** Representative fluorescent turn-on G4 ligands and their corresponding characteristics and applications

Class	Ligand and Commercial Availability (CAS no.)	Structure and Fluorescence Properties	Representative Applications	Advantages and Limitations	Ref.
Porphyrin	*N*-methyl mesoporphyrin IX (NMM), Yes (142234-85-3)	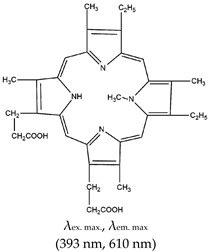	— Highly specific parallel telomeric G4s binding, stabilization, and structural rearrangement. — Specific inhibitor of G4 unwinding by helicase (of *Saccharomyces cerevisiae* and human BML). In the presence of NMM the helicase gets trapped on the NMM-G4 complex. — Real time specific G4 based fluorescence assay for RNase sensing and inhibition. — A label free sensor for sensing iodide and melamine. Based on thymine-melamine-thymine (T-M-T)/thymine-Hg^2+^-thymine (T-Hg^2+^-T) and G4-NMM complex. — Highly sensitive microbial pathogen sensor based on quaternized magnetic nanoparticle exonuclease III assisted DNA amplification assays. Based on conformational transition from hairpin to G4 (assisted by Exo III nuclease) and subsequent specific interaction (of the G4) with NMM.	— Asymmetric anionic porphyrin — Inhibitors of different enzymes — Easy to develop label free sensors — Selective and sensitive G4 ligand — Allow real time study of G4 — Specific binding to parallel telomeric G4 — Microbial pathogen sensing — In live cell imaging, a Stokes shift and a red-shift emission were observed when applied, both of which were higher than the emissions seen with a different class of ligand, thioflavin T (ThT) — Not shown to provide visual discrimination of various G4s	[75,76,79,84,146,147]
	5,10,15,20-tetra-{4-[2-(1-methyl-1-piperidinyl)ethoxy]phenyl porphyrin (TMPipEOPP), No	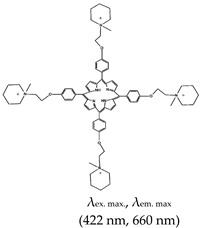	— G4s specific probe that allow visual discrimination between G4s, duplexes, and single stranded DNAs.	— Cationic porphyrin — Aid visual differentiation of various G4s — Not shown to have enzymatic potentials	[86]
	Pyridinium, 4,4′,4′′,4′′′-(21*H*,23*H*-porphine-5,10,15,20-tetrayl)tetrakis[1-methyl-, 4-methylbenzenesulfonate (1:4) (TMPyP4), Yes (36951-72-1)	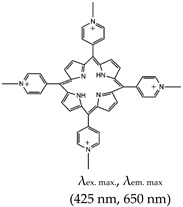	— A ligand specific metabolic regulation of nitrate assimilation in *Paracoccus denitrificans* (a Gram-negative soil bacterium), based on stabilization of G4.	— Cationic porphyrin — Metabolic regulator nitrate assimilation — Not shown to provide visual discrimination of various G4s. — Not shown to have inhibitory potentials	[85]
Benzothiozole	3,6-dimethyl-2-(4-dimethylaminophenyl) benzothiazolium cation Thioflavin T (ThT), Yes (2390-54-7)	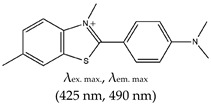	— Sensitive and efficient G4 fluorescence sensor for human telomeric DNA. — Fluorescence sensor for the determination of cysteine and glutathione. Also, shown to detects biothiols in blood samples. Based on ThT ability to induced conformational specific G4. — Highly sensitive sensor for thrombin detection using Förster resonance energy transfer (FRET). Based on ThT ability to induce G4 and used as an acceptor with a conjugated polymer as the donor. — Specific fluorescence probe for G4 formation. Based on direct interaction between ThT and the target. — Aptasensor for Adenosine deaminase activity and inhibition. — Fluorescence probe for the detection of G4s in *Chlamydomonas reinhardtii.* *—* Fluorescence probe for the detection of G4s in zika virus. — Fluorescence probe for the detection of G4s in papillomaviruses.	— Has low background fluorescence intensity, which translates to a high signal-to-noise ratio — Highly sensitive probe for the detection of G4s in different species and in different samples — Induced conformational specific G4 — Shown to have enzymatic activity and inhibition — An ethyl substituted ThT allows naked-eye visualization of G4 in solution under ultraviolet light — Highly specific to parallel G4s — In live cells imaging, it produces less emissions compared to what was observed with a different class of ligand, NMM — In live cell imaging, its green fluorescence can easily coincide with the intrinsic fluorescence of the cell’s other components — ThT induced G4s can potentially cause topological changes — Produces false positive and false negative results — It binds tightly to non-G4 G–A-rich containing sequences and dimerizes them into a parallel double-stranded mode — Difficult to use for effective monitoring of G4s in the chromatin of live cells because of its inability to stain the nuclei	[72,74,97,99,104,105,106,110,148]
	*N*-Isopropyl-2-(4-*N*,*N*-dimethylanilino)-6-methylbenzothiazole (IMT), No	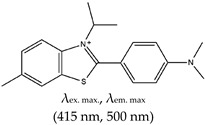	— Real time fluorescence probe for monitoring the formation of G4 in live cells and its response to chemical treatment demonstrated.	— Live cell monitoring of G4 formation in real time — Selectively bind G4s in a cell’s chromatin (with negligible cytotoxicity) — Toxicity analysis only performed using single method instead of using two different methods in parallel	[51]
	ThT-NE, No	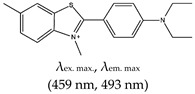	— Cell permeable and highly specific G4 based fluorescence turn-on probe for real time imaging of native viral RNA genome in hepatitis C virus (HCV). This method was shown to allow subcellular monitoring and continuous live-cell monitoring of infected cells.	— Allows real time subcellular and continuous live-cell monitoring of native viral RNA genome — Toxicity effect to cells not shown/reported	[107]
Triphenylmethane (TPM)	Crystal Violet (CV), Yes (548-62-9)	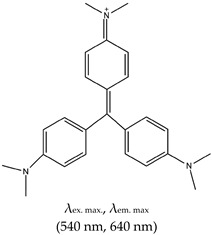	— Label free fluorescence aptasensor for specific detection of CV based on G4 interaction with CV. — A label-free G4 based fluorescence turn-on probe for the selective detection of ATP in aqueous medium. This is based on the ability of CV to specifically binds to G4. — Live cells visualization of G4 role in alternative splicing via RNA-binding protein hnRNPF. — G4-based fluorescence aptasensor for the selective detection of thrombin protein. Based on CV-G4 fluorescence. — Fluorescence probe for monitoring G4 structural differences (as a function of cation) and sensing of K^+^. — Fluorescence probe for homogenous detection of K^+^ based on the fluorescence intensity changes of CV-G4 complex.	— Distinguishes intramolecular from intermolecular G4s — Distinguishes single DNA strands from duplex DNAs — Widely employed in biosensing — Distinguishes between parallel and anti-parallel G4 topologies (preferentially binds to anti-parallel G4s)	[73,116,117,118,119,120]
	Malachite Green (MG), Yes (569-64-2)	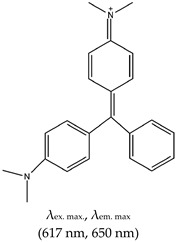	— Fluorescence G4 based aptasensor for binding recognition to MG ligand.	— Widely employed in (bio)sending — Not shown to allow naked eye visualization of G4s in solution	[146]
Triangulenium	Morpholino containing bis-substituted triangulenium (DAOTA-M2), **No**	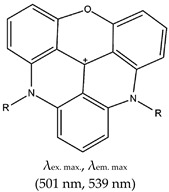	— Fluorescence probe for G4 visualization in live cells, based fluorescence lifetime imaging microscopy. This probe was demonstrated to be cell permeable, have low toxicity, and be localized in the nucleus.	— Allows live cell visualization of G4 — High cell permeability — Low cytotoxicity — Can localize in the nucleus — One-to-one G4-specific sensor — Allows the visualization of interactions between ligands and G4s by fluorescence lifetime microscopy	[52]
Imidazole	Ethyl 2-(6-(4-(4-((4-(4,5-bis(4-(4-methylpiperazin-1-yl)phenyl)-1*H*-imidazol-2-yl)phenoxy)methyl)-1*H*-1,2,3-triazol-1-yl)butoxy)-3-oxo-3*H*-xanthen-9-yl)benzoate (IZFL-2), No	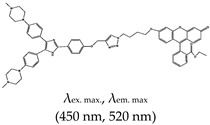	— A tunable fluorescence activation probe for the specific detection of c-Myc G4. This was demonstrated to differentiate between wild-type c-Myc G4 and other G4s.	— Its fluorescence can be tuned — Distinctive smart sensor specific only for c-Myc G4s — Not yet demonstrated in live cells	[139]
	2,4,5-triaryl-substituted imidazole (IZCM-1), No	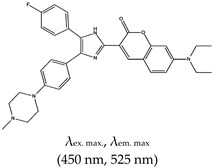	— Fluorescence turn-on probe for the specific detection of parallel G4 without affecting their topology and thermal stability.	— Effectively and specifically binds to parallel G4s	[137]
	[2-(4-(4,5-bis(4-(4-methylpiperazin-1-yl)phenyl)-1*H*-imidazol-2-yl)phenyl)-6-(4-methylpiperazin-1-yl)-1*H*-benzo[de]isoquinoline-1,3(2*H*)-dione] (IZNP-1), No	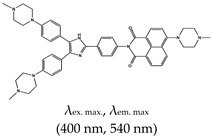	— Fluorescence turn-on probe for the specific targeting of telomeric multimeric G4 structures, shown to occur via intercalation into the pocket between two G-quartet units.	— Can discriminate between telomeric multimeric G4s and monomeric G4s— Induce apoptosis and senescence in cancer cells	[138]
Acridine	3,6,9-trisubstitutedAcridine; cyanine dye 1, No	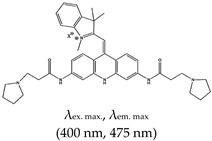	— Water soluble dual function probe for G4 specific binding; pH sensitive and fluorescence probe for G4 stabilization and detection that operate by a push–pull mechanism.	— Highly water soluble — pH sensitive — Application not demonstrated in vivo	[133]
Alkaloid	(*E*)-3-((7-(diethylamino)-2-oxo-2*H*-chromen-3-yl)methylene)-6,7-difluoro-4-methyl-9-oxo-1,2,3,9-tetrahydropyrrolo[2,1-*b*]quinazolin-4-ium iodide (ISCH-1), No	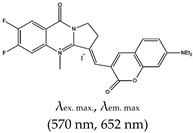	— Multifunctional (colorimetric and red-emitting fluorescence) turn-on probe for specific G4 detection. This method is ideal for reliability and diverse applications.	— Multifunctional (colorimetric and fluorescence) — Reliable and potential for numerous applications — Not shown to allow specific targeting of G4s in a given region (such as the 5′-UTR)	[141]
	(*E*)-3-((7-(diethylamino)-2-oxo-2*H*-chromen-3-yl)methylene)-7-fluoro-4-methyl-9-oxo-6-(prop-2-yn-1-yloxy)-1,2,3,9-tetrahydropyrrolo[2,1-*b*]quinazolin-4-ium (ISCH-oa1), No	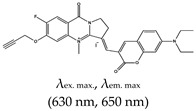	— G-quadruplex-triggered fluorogenic hybridization (GTFH) probe, that selectively allows the visualization of the G-quadruplexes that form in a particular region interest (NRAS mRNA 5′-UTR region was demonstrated) both in vitro and in cells. The ligand consists of two segments, which are a fluorescent light-up fluorophore and oligonucleotide sequence that can hybridize with the sequence adjacent to the guanine rich sequence in the NRAS mRNA 5′-UTR or other regions of interest.	— Allows specific targeting of G4s in a particular region such as 5′-UTR — Can be use both in vivo and in vitro — Cannot detect the in situ spots of a given RNA in single cell — Requires RNAs to be transfected into cells to increase their concentration	[142]
	(*E*)-2-(2-(7-(diethylamino)-2-oxo-2*H*-chromen-3-yl)vinyl)-6-fluoro-1-methyl-7-(4-methylpiperazin-1-yl)quinolin-1-ium iodide (QUMA-1), No	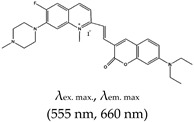	— Highly selective fluorescence turn-on probe for real time and continuous tracking and monitoring of rG4 structural dynamics in live cells, this application has been demonstrated in through live cell imaging. Also, applied in visualization of rG4s unwinding by helicase.	— Allows live cell monitoring and tracking of rG4s — Allows the imaging of rG4 unwinding — Fluorescence intensity decreases in the presence of competing G4s ligands	[143]
Acetone	Bis(4-aminobenzylidene)acetone derivative referred to as GD3, No	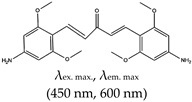	— An effective red emitting fluorescence turn-on ligand for parallel G4s structures. Its biological application was demonstrated in fixed cells and shown to allow the visualization and monitoring of G4s structures. It was also shown to occur based on dipole moment created in the microenvironment of the ligand and restriction of the fluorophore resulting in altered charge transfer in the system, hence an enhanced light-up observed	— Red emitting ligand — Specific for parallel G4s — Allows monitoring of G4s in fixed cells	[136]
Pyrene	Pyrene template-assembled synthetic G-quartet (PyroTASQ), No	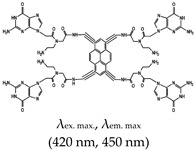	— Multitasking G4s smart probe (stabilizing ligand and fluorescence turn-on probe). This ligand and probe were demonstrated to recognize and bind to both DNA and RNA G4s, and shown to occur through an interesting approach; in which the ligand causes a ‘quadruplex-promoted conformational switch’ that leads to assembling of four guanines into a G-quartet, and subsequently the pyrene’s fluorescence is release	— Multifunctional (stabilization and fluorescence turn-on) — Can bind to both DNA and RNA G4s — Failed for in vivo studies as it forms aggregates in cells	[144]
Naphthalene	Naptho-template-assembled synthetic G-quartet (N-TASQ), No	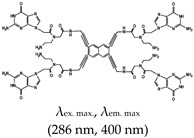	— Multitasking G4s smart probe stabilizing ligand and fluorescence turn-on probe for live cell visualization of RNA G4s using multi-photon microscopy technique, while both RNA and DNA G4s were visualized using confocal microscopy. The interaction occurs through similar approach with Pyro-TASQ.	— Multifunctional (stabilization and fluorescence turn-on) — Can bind to both DNA and RNA G4s — G4 visualization in live cells using the multi-photon microscopy — Allows both RNA and DNA G4 imaging using confocal microscopy — No binding competition with other G4 ligands shown	[108,145]

**Table 2:** Representative list of G-quadruplex-containing nucleic acid aptamers.

Aptamer	Target	Sequence (5′-3′)	Length	Ref.
**DNA G-quadruplex-containing aptamers**				
TBA	Thrombin	d(GGTTGGTGTGGTTGG)	15	[156]
AS1411	Nucleolin	d(GGTGGTGGTGGTTGTGGTGGTGGTGG)	26	[185]
T40214	Stat3 ^a^	d(GGGCGGGCGGGCGGGC)	16	[190]
T40231	Stat3	d(GGTGGGTGGGTGGG)	14	[190]
22AG	Human TEBPs ^b^	d(AGGGTTAGGGTTAGGGTTAGGG)	22	[95]
*N.A.*	Ciliate TEBPs	d(TTTTGGGGTTTTGGGG)	16	[191]
ISIS5320	HIV-1 gp120	d(TTGGGGTT)	8	[192]
93del	HIV-1 integrase	d(GGGGTGGGAGGAGGGT)	16	[182]
112del	HIV-1 integrase	d(CGGGTGGGTGGGTGGT)	16	[183]
T30695	HIV-1 integrase	d(GGGTGGGTGGGTGGGT)	16	[182]
RT5	HIV-1 reverse transcriptase	d(CAGGCGCCGGGGGGGTGGGAATACAGTGATCAGCG)	35	[41]
RT6	HIV-1 reverse transcriptase	d(CAGGCGTTAGGGAAGGGCGTCGAAAGCAGGGTGGG)	35	[41]
RT47	HIV-1 reverse transcriptase	d(CAGGCCTTGGGCGGGCCGGGACAATGGAGAGATTT)	35	[41]
ODN93	HIV-1 reverse transcriptase	d(GGGGGTGGGAGGAGGGTAGGCCTTAGGTTTCTGA)	34	[193]
r10/43.	HCV RdRp ^c^	d(GGGCGTGGTGGGTGGGGTACTAATAATGTGCGTTTG)	36	[194]
G5	SARS Coronavirus Helicase	d(AGCGGGCATATGGTGGTGGGTGGTATGGTC)	30	[195]
*N.A*.	Insulin	d(GGTGGTGGGGGGGGTTGGTAGGGTGTCTTC)	30	[196]
*N.A.*	Hematoporphyrin IX	d(ATGGGGTCGGGCGGGCCGGGTGTC)	24	[197]
PS2M	Hemin	d(GTGGGTAGGGCGGGTTGG)	18	[198]
ABA	ATP	d(ACCTGGGGGAGTATTGCGGAGGAAGGT)	27	[167]
**RNA G-quadruplex-containing aptamers**				
Spinach	DFHBI ^d^	r(GACGCAACUGAAUGAAAUGGUGAAGGACGGGUCCAGGUGUGGCUGCUUCGGCAGUGCAGCUUGUUGAGUAGAGUGUGAGCUCCGUAACUAGUCGCGUC)	98	[175]
Spinach mini	DFHBI	r(GACGCGACCGAAAUGGUGAAGGACGGGUCCAGUGCUUCGGCACUGUUGAGUAGAGUGUGAGCUCCGUAACUGGUCGCGUC)	80	[175]
Spinach1.2	DFHBI	r(GACGCGACCGAAUGAAAUGGUGAAGGACGGGUCCAGCCGGCUGCUUCGGCAGCCGGCUUGUUGAGUAGAGUGUGAGCUCCGUAACUGGUCGCGUC)	95	[178]
Spinach2	DFHBI	r(GAUGUAACUGAAUGAAAUGGUGAAGGACGGGUCCAGUAGGCUGCUUCGGCAGCCUACUUGUUGAGUAGAGUGUGAGCUCCGUAACUAGUUACAUC)	95	[178]
Spinach2 mini	DFHBI	r(GAUGUAACUGAAAUGGUGAAGGACGGGUCCAGUGCUUCGGCACUGUUGAGUAGAGUGUGAGCUCCGUAACUAGUUACAUC)	80	[179]
Baby Spinach	DFHBI	r(GGUGAAGGACGGGUCCAGUAGUUCGCUACUGUUGAGUAGAGUGUGAGCUCC)	51	[180]
Broccoli	DFHBI	r(GAGACGGUCGGGUCCAGAUAUUCGUAUCUGUCGAGUAGAGUGUGGGCUC)	49	[199]
Corn	DFHO ^e^	r(CGAGGAAGGAGGUCUGAGGAGGUCACUG)	28	[200]
Mango	Thiazole orange-biotin	r(GGCACGUACGAAGGGACGGUGCGGAGAGGAGAGUACGUG)	39	[109]
Mango-II	Thiazole orange-biotin	r(GCGUACGAAGGAGAGGAGAGGAAGAGGAGAGUACGC)	36	[201]
Mango-III	Thiazole orange-biotin	r(GCUACGAAGGAAGGAUUGGUAUGUGGUAUAUUCGUAGC)	38	[202]
ApT4-A	Thyroxine hormone	r(GGUGGAGGGGGACGUGCUGCAUCCGCAGUGCGUCUUGGGUUGUG)	44	[203]
*N.A.*	Human receptor activator of NF-*k*B	r(ACGGAUUCGUAUGGGUGGGAUCGGGAAGGGCUACGAACACCGU)	43	[204]
*N.A.*	HIV-1 integrase	r(GGAGGGAGGGGAU) or r(GGAGUUAGGGGCU)	13	[205]
*N.A.*	Prion protein rPrP23-231	r(CACUGCUACCUUAGAGUAGGAGCGGGACGAGGGGUUGUUGGGACGUGGGUAUGAUCCAUACAUUAGGAAGCUGGUGAGCUGGCACC)	86	[206]
N2	Trypanosome	r(AAGAAGCGCGCGAGGCAGGACGAGGCAGGUGAGCGCUGUCCGA)	43	[207]

^a^, Signal transducer and activator of transcription 3; ^b^, Telomere end-binding proteins; ^c^, RNA-dependent RNA polymerase; ^d^, 3,5-difluoro-4-hydroxybenzylidene imidazolinone; ^e^, 3,5-difluoro-4-hydroxybenzylidene-imidazolinone-2-oxime.
